# The Real and Relative Value of Our Recent Antipyretics

**Published:** 1891-05

**Authors:** J. C. Johnson

**Affiliations:** Atlanta, Ga., Lecturer on Diseases of Children, Atlanta Medical College


### Original Communications.

### THE REAL AND RELATIVE VALUE OF OUR RECENT ANTIPYRETICS.*

BY J. C. JOHNSON, M. D., ATLANTA, GA.,

Lecturer on Diseases of Children, Atlanta Medical College.

Perhaps in no era of medicine, since it has attained the dignity of a science, have so many rival remedies sought recognition in materia medica as are now clamoring for use. Scarcely have the merits of one been adjudged, and its place in therapeutics assigned, before its successor, boasting superior virtues, is announced and accepted, only to meet a similar fate. Thus the list of remedies lengthens, but to prove the narrow range of applica-

*Read before the Atlanta Society of Medicine.

tion and uncertainty of them all, reflecting discredit upon this branch of medicine, and inviting the introduction into popular practice of numberless proprietary preparations and patent nostrums whose only benefit is the tax they pay.

There is a measure of responsibility inseparable from our support of corporations whose high office is to prescribe in certain forms and combinations certain drugs for our profession to dispense. Progress in therapeutics is attained by individual observation and research, and not by the general and ready acceptance of advertised opinions.

Our object is to mitigate suffering and cure disease, and it were criminal folly to reject a remedy, of established efficacy, simply because we did not know its method of operation. Digitalis should be employed in valvular diseases of the heart, had experience only proved that it accomplishes the end desired, but its greater usefulness rests in the fact that it does so by lengthening diastolic and strengthening systolic action.

It is not my purpose to decry the worthy attempts of pharmacists, nor repel the advent of any agent which promises greater efficacy or exactness in therapeutics. With increasing and important developments in the various fields of pathology, we need equal advance in therapeutics, and we can never determine the merits of the new while we exclusively employ the old; but when on uncertain seas, let us not forget our unvarying compass, lest while steering from Scylla we are engulfed by Charybdis.

In composing this paper, my chief purpose was to place before the society for discussion some of our most recent remedies. My knowledge of and experience with them are too limited to encourage the hope of enlightening you upon their merits or modes of action. But for our own mutual good, and especially for my own gratification, I would evoke expressions from the members, to determine and agree upon their utility and indications for employment, in the light of our present knowledge concerning them.

I refer to antipyrin, phenacetin, antifebrin and acetanilid. We all know that the last two are one and the same, antifebrin being the trade name for the compound more properly called acetanilid. Antipyrin was discovered in 1884, by Dr. Ludwig

Knorr, of the University of Wurzburg, Bavaria, in an attempt to make quinine synthetically.

Antifebrin was prepared as a chemical by Gerhardt, in Germany, in 1852, but its use as a medicinal agent is of comparatively recent date.

Phenacetin, the last addition to this number, has been in use a shorter period than either of the others, though it has quickly won its way as their successful rival.

Chemically speaking, all these are of the coal tar series, are unstable, and cannot be given with acids or acid salts. Strong alkalies decompose them. Neutral salts are compatible with them. Phenacetin is the most stable of them all, and can be combined with a larger number of chemicals than the others. It is not decomposed by dilute nitric or hydrochloric acid. It is also the least soluble of them all. Antipyrin is easily soluble in less its weight in water, and I most frequently prescribe it with syrup of tolu. Antifebrin, or acetanilid, is less soluble than antipyrin, but can be conveniently mixed with whiskey. Either of these three can now be had in the form of tablets.

It is with the therapeutical action of these drugs that we are chiefly concerned. Their first claim was as antipyretics, but later observation proved also their ability to relieve pain. These two effects are generally conceded them now. I do not know that any curative power in any disease has been accorded them or, if so, that it has been established. It would greatly aid us, in our considerations, and enhance the interest of the paper to quote some popular opinions touching upon the pathology of fever, that we might look from cause, through its operation, to effect, but time does not permit, and I will only mention a few facts which force themselves into notice along the way.

The virtue of a drug is best determined by its combined action compared to other agents of the same class, in the accomplishment of a given result. How, then, do these medicines compare with others longer used and of established efficacy? Granting the necessity or expediency of reducing the temperature in a given disease, that agent is best which soonest and safest serves that end, and which at the same time least disturbs the other functions of the body, physiologically performed, and aids in removing the cause or checking the progress of the disease upon which the elevated temperature depends.

The part which the nervous system plays in the production of fever is not yet fully decided ; though, in addition to the result of experiments bearing upon this question, the pathology of certain diseases, attended by characteristic elevation of temperature, proves the intimate relation between the accompanying fever and the impress of its cause upon the nervous system, though hardly determining the primary or secondary nature of either. Should the cause originate *de novo* in the nerve centers as appears in states of overwork, worry and exhaustion, or did the exciting agent manifest its impress through the intervention of the nerves that remedy wouldbe most curative which directly, by stimulative, or sedative, or alterative action, restrained or neutralized its effect,

The *nidus* of irritation in inflammatory fever is evidently the phenomena of perverted action, the result of injury, whether that injury arises from external violence, or a poison without, or is the result of morbid material generated within the system. What best reduces the amount of irritation, whether by direct influence or by destroying the source, or checks the development or progress of the inflammatory process, is our most valuable and suitable antipyretic. Hence no agent, without positive action upon the heart, or influence upon arterial tension and the great sensorium as well, can successfully rival opium, veratrum and aconite in the treatment of this form of fever.

In this age, no one would attempt to cure malarial fevers without quinine or some form of cinchona. Neither of the remedies under discussion prevents a chill or an exacerbation. But in the continued type, one of them—preferably phenacetin—may be added for prompter and results more grateful to the feelings of the patient.

In self-limiting diseases we do not hope to check their course by reducing the fever ; and it has been questioned how far we are justifiable in interfering with this symptom, yet the doctor of today would not be held blameless who neglected this feature of treatment, and we owe it to our patient, as well as ourselves, to bring and maintain the various functions of the body as near their normal state as possible, or direct their action to a condition which we think best preserves the vitality of the system and hastens convalescence. Hence in diseases of debility, as typhoid fever, the question arises, are we by heroic measures to disturb its even course, or regarding it as a bridge supplied by nature to sustain the patient while all his forces are engaged in the active and uncertain contest with disease and death, withhold those agents which only attack the fever and leave its cause unmolested? Obviously it is better to reinforce than to supplant nature in her efforts to throw off the fetters of the enemy.

Her manner of doing this is seen in the malarial chill. So the spirit of mindererus or spirit of nitrous ether deserves preference over either of our new antipyretics in diseases of this class. Though I have had speedy and happy results from phenacetin when the aforesaid remedies had failed upon fair and extended trial. No one now disputes the antipyretic action of these drugs. They unmistakably effect the end for which they are given. The only question is, when they are indicated and which is to be preferred. Some think that antipyrin is the most reliable of them all. I favor and almost exclusively prescribe phenacetin. The only difference between antifebrin and acetanilid is in their cost, which is considerable.

But these remedies claim another besides the effect mentioned, and in this, perhaps, vary more not only in extent but in their seat of action.

Antipyrin impresses chiefly and almost solely the sensory matter of the cerebro-spinal system. Its most decided effect is upon the brain, and is one of our best remedies for headaches and neuralgias of the face. Its anodyne effect diminishes as it is removed from the head, or as the cause of the pain in the head is distant from it. I have never witnessed any positive action on the special senses or motor system, nor have I ever seen stupor produced by it even in the largest doses. I recall a case of rheumatism of long standing in which I pushed the drug to thirty grain doses every two or three hours, not even drowsiness resulting. While its efficacy is not to be compared to a hypodermic injection of morphia yet when relief from pain is the end desired, and where stupefaction is to be avoided, or where opium is contraindicated by idiosyncrasy or fear of habit, antipyrin may be employed with great advantage.

The effect of phenacetin on the sensory apparatus is more general and save about the head, equally as decided as that of antipyrin. Perhaps no remedy was ever more liberally and universally employed than was phenacetin during the recent epidemic, of La Grippe which visited our country. Its independent adoption in the treatment of this disease stands as proof of the merit which it claims. In this, as you know, it acted most happily, reducing the fever and allaying pain, though it did not check the progress of the disease nor prevent the results. I observed, too, that the pain which followed La Grippe yielded more readily to salicylate of soda than to phenacetin.

Its prompt relief of the asthmatic symptoms which generally were present in La Grippe, as well as the terrible tormina of the bowels, lead me to hope that this apparent anti-spasmodic property might be applied in the treatment of other diseases, if not with kindred pathology, in which the same system of nerves was involved. I have since used it in asthma and was disappointed in the result, the patient complaining that her distress was intensified after taking it, and that she experienced more difficulty in expectoration.

I have never known death to be produced by either of these drugs, though it was supposed that a combination of calomel and antipyrin caused the death of a child in New York. Antipyrin is not as free from danger as antifebrin and phenacetin. Not a few cases of heart failure are reported as following its use. I have witnessed this effect only once in my own practice—where ten grains were given. In the case of rheumatism before alluded to, where it was given in enormous doses, there were no alarming symptoms, and none unfavorable excepting slight gastric and intestinal irritation.

Why and exactly how it depresses the heart's action, I think still remains unknown. The evidence is in favor of the supposition that it impresses chiefly and primarily the resident ganglia of the heart. There is no disturbance of the other organs supplied by a common centre, which cannot be explained by the intimate relation and interdependence of their functional action, excepting it be that on the gastro-intestinal canal, which is either irritant locally, or the result of disturbance in the ganglionic fibres. But the fact remains and should be observed in its employment.

I have never heard of a similar effect following phenacetin, and since it has virtues equal to those of antipyrin, and is cheaper, I consider it preferable for general use.

Antifebrin sometimes produces prostration in an extreme degree, and is no more reliable than phenacetin as an antipyretic, and less than antipyrin as an anodyne. I use the chemical form when I prescribe it, especially for the sake of my poorer patients.

Therapeutics could have dispensed with these agents, but their undisputed merits have won them a welcome into our list of remedies, leaving their exact and relative rank to be determined by their more extended use.

